# Multiple entry pathways within the efflux transporter AcrB contribute to multidrug recognition

**DOI:** 10.1038/s41467-017-02493-1

**Published:** 2018-01-09

**Authors:** Martijn Zwama, Seiji Yamasaki, Ryosuke Nakashima, Keisuke Sakurai, Kunihiko Nishino, Akihito Yamaguchi

**Affiliations:** 1 Laboratory of Cell Membrane Structural Biology, Institute of Scientific and Industrial Research, Osaka University, Ibaraki, Osaka 567-0047 Japan; 2 Department of Biomolecular Science and Regulation, Institute of Scientific and Industrial Research, Osaka University, Ibaraki, Osaka 567-0047 Japan; 3 Graduate School of Pharmaceutical Sciences, Osaka University, Suita, Osaka 565-0871 Japan

## Abstract

AcrB is the major multidrug exporter in *Escherichia coli*. Although several substrate-entrances have been identified, the specificity of these various transport paths remains unclear. Here we present evidence for a substrate channel (channel 3)  from the central cavity of the AcrB trimer, which is connected directly to the deep pocket without first passing the switch-loop and the proximal pocket . Planar aromatic cations, such as ethidium, prefer channel 3 to channels 1 and 2. The efflux through channel 3 increases by targeted mutations and is not in competition with the export of drugs such as minocycline and erythromycin through channels 1 and 2. A switch-loop mutant, in which the pathway from the proximal to the deep pocket is hindered, can export only channel 3-utilizing drugs. The usage of multiple entrances thus contributes to the recognition and transport of a wide range of drugs with different physicochemical properties.

## Abstract (web summary)

Multidrug transporters possess several drug binding sites. Here the authors describe a transport path specific for planar aromatic cations in the *E. coli* multi-drug transporter AcrB.

## Introduction

Antibiotic resistance in pathogens forms a threat to global health, as bacteria have been developing mechanisms to undermine the effects of several to all classes of antibiotics commercially available today^1–6^. In Gram-negative bacteria, one of the main mechanisms of multidrug resistance is the overexpression of intrinsic tripartite efflux pump complexes spanning the inner and outer membranes^7^. These complexes comprise an inner membrane efflux transporter (e.g. AcrB), an outer membrane protein (e.g. TolC), and a periplasmic membrane fusion protein (e.g. AcrA)^3, 8, 9^.

The understanding of drug efflux by multidrug transporters belonging to the Resistance-Nodulation-Division (RND) family has significantly evolved over time. From the initial crystal structure of an RND efflux transporter (AcrB), a homotrimer with a three-fold symmetry^10^, it was hypothesized that drugs translocate through the vestibule to the central cavity and then up through the central pore in the AcrB trimer. This hypothesis was modified based on a drug-bound structure of AcrB in its physiologically active state^11^. Based on this asymmetrical crystal structure, we proposed the functionally rotating mechanism, with three distinct monomer conformations: access, binding and extrusion (or: loose (L), tight (T) and open (O)). Additionally, an alternative drug extrusion pathway was suggested where drugs translocate from the membrane surface channel entrance (located next to the vestibule), and was later called channel 1 (CH1). From this channel, drugs can move to a voluminous multisite drug-binding pocket within the AcrB monomer, from where the drugs are extruded through a top-open funnel^11^. Later, an additional periplasmic entrance named channel 2 (CH2), was identified^12^. The asymmetric structure and dual entrances are also confirmed in related RND transporter MexB^13^.

An additional multisite drug-binding pocket was found, which binds high-molecular-mass drugs (HMMDs) in the AcrB access monomer. This binding pocket was named the proximal binding pocket (PBP)^14^. The PBP is tandemly placed before the previously found low-molecular-mass drugs (LMMDs) binding pocket^11^, which was renamed as the distal binding pocket (DBP). A glycine-rich swinging loop is located between the PBP and DBP. This loop acts as a swinging valve during the translocation of drugs from the PBP to the DBP by a peristaltic motion^14^. We previously suggested the presence of a third channel (CH3) open to the central cavity within the AcrB trimer. CH1, CH2 and CH3 were all hypothesized to be merged at the PBP^14^. A similar third entrance channel was also pointed out by others^15, 16^. The importance of CH1 and CH2 is revealed by site-directed mutagenesis studies^14^, while there was yet no experimental evidence for CH3. Multiple substrate binding sites and the multiple entrances were later confirmed^17^ and the importance of the swinging loop between the PBP and the DBP was also identified . This loop was named the switch-loop^18^.

It therefore appeared that RND-type exporters have multiple drug-binding pockets as well as multiple drug translocation pathways. However, the significance of the existence of multiple entrances had not been clearly established. Here, we provide evidence for a specific  entry channel from the central cavity and show that this channel (CH3) is directly connected to the DBP and bypasses the PBP and the switch-loop. The channel is present in the binding monomer of AcrB. We show that CH3-preferring drugs can be characterized by their chemical structure and are transported non-competitively with the other CH1 and CH2-preferring drugs.

## Results

### Effect of central cavity mutations on efflux ability of AcrB

**Fig. 1:**
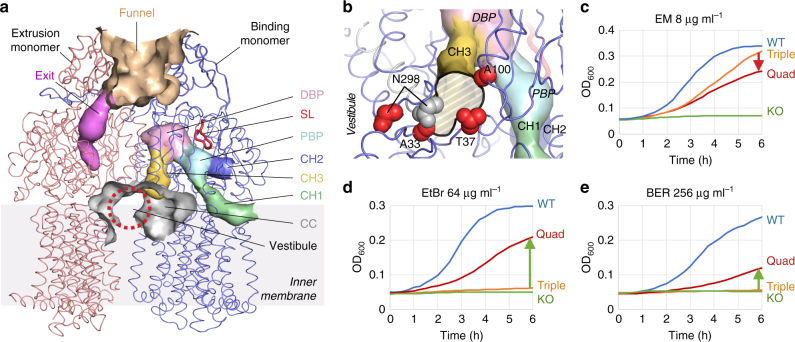
Channels within AcrB and the effect of central cavity channel mutations on transport activity. **a** The three main supposed channels for AcrB are CH1 (green), CH2 (blue) and CH3 (yellow) drawn by CAVER^23^ in the binding monomer. The access monomer in the front is deleted for a clearer view on the extrusion and binding monomers, their channels and the central cavity. Although the originally postulated CH3^14, 16^ was connected to the PBP, here we show that the new more likely CH3 has a common entrance with the original CH3, but is directly connected to the DBP, bypassing the PBP and the switch-loop. The vestibule is shown by a red dashed circle and the central cavity is shown as a grey cave. For this figure, we used PDB 3AOD, although the channel is visible in other crystal structures of the AcrB protein. DBP distal binding pocket, PBP proximal binding pocket, SL switch-loop, CH1, CH2 and CH3, channels 1, 2 and 3, respectively; CC central cavity. **b** Rotated and magnified view from the central cavity on the CH3 entrance. Red spheres indicate the mutation sites used in this study, depicted in the binding monomer. The grey spheres show superimposed N298 in the access monomer, where it is located near the CH3 opening. The opening is depicted as a black outlined white/grey area. **c**−**e** Growth ability of *E. coli* MG1655Δ*acrB* expressing various CH3 mutants supplemented with erythromycin (EM) (8 μg ml^−1^) (**c**), ethidium bromide (EtBr) (64 μg ml^−1^) (**d**) and berberine (BER) (128 μg ml^−1^) (**e**). Colours blue, orange, red and green indicate wild-type AcrB, A33W/T37W/N298W triple mutant, A33W/T37W/A100W/N298W quadruple mutant, and vector only, respectively. Green arrows mark an increase in activity, red arrow marks a decrease in activity for quadruple compared to triple mutant AcrB. A complete overview of MIC values for the mutants can be found in Tables 1 and 2 and growth curves with other concentrations can be found in Supplementary Fig. 4

We previously suggested three possible entrance channels of AcrB: open to the surface of the cell membrane (CH1), open to the periplasm (CH2) and open to the central cavity (CH3) (Fig. 1a) and that multiple entrances might contribute to extend the substrate specificity^14^. Among these channels, only CH3 lacks experimental evidence. Here, we introduced Trp mutations at the positions Ala33, Thr37, Ala100 and Asn298, which are located around the entrance of CH3, connecting to the central cavity of the AcrB trimer (Fig. 1b). In the binding monomer, only Asn298 swings away significantly from the CH3 entrance towards the vestibule. Table 1 shows the effect of these single or combined mutations on agar plate minimum inhibitory concentration (MIC) values for 13 different drugs and toxic compounds. Expression levels of these mutants were similar to wild-type AcrB (Supplementary Fig. 1). No single mutation affected the MIC values, except for N298W. The N298W mutation caused a one or two dilution step decrease in MIC for most of the drugs. Because position 298 is one of the most mobile residues during functional rotation (Fig. 1b), the introduction of a bulky tryptophan residue probably inhibits efficient movement of the protein. The N298W mutation causes a partial (thus not complete) inhibition in export activity, showing that the functionally rotating mechanism is still possible. Although the A33W/T37W double mutations did not affect the MICs, the triple mutation (A33W/T37W/N298W) caused an additional one dilution step decrease of the MICs compared to N298W. This is probably because the A33W/T37W double mutations alone may be masked in wild-type AcrB’s high level of activity, while the effect may become apparent under the limited activity of the N298W mutant.

**Table 1:** MIC values for channel 3 (CH3) mutant AcrB-expressing *E. coli* MG1655Δ*acrB* cells

Strains and mutations	Minimal inhibitory concentration (MIC, µg ml^−1^)												
	PAC					LMMD					HMMD		
	BZK	R6G	EtBr	ACR	CV	MINO	CLX	DEQ	ENO	NOR	NB	EM	RIF
MG1655∆*tolC*	4	32	8	8	2	0.25	0.25	4	0.0625	0.0078125	1	2	8
MG1655∆*acrB*	**4**	**32**	**8**	**16**	**2**	**0.25**	**1**	**4**	**0.0625**	**0.015625**	**4**	**2**	**8**
Wild-type AcrB	*256*	*8192*	*1024*	*1024*	*16*	*2*	*256*	*512*	*0.25*	*0.0625*	*1024*	*128*	*8*
T37W	256	8192	1024	1024	16	2	512	512	0.125	0.0625	1024	256	8
A100W	256	8192	1024	1024	16	2	256	512	0.125	0.0625	1024	128	8
N298W	64	512	256	32	16	2	128	128	0.125	0.03125	256	128	4
A33W/T37W	256	8192	1024	512	16	2	512	256	0.125	0.0625	1024	128	16
Triple (A33W/T37W/N298W)	32	256	128	32	8	1	32	64	0.0625	0.03125	256	64	4
Quadruple (A33W/T37W/A100W/N298W)	64	512	512	64	8	1	32	64	0.0625	0.015625	128	32	4

MIC values are measured by a twofold dilution method on agar plates as mentioned in Methods. Values are in µg ml^−1^. Bold values highlight the MIC values for KO cells. Italic values highlight the MIC values for wild-type AcrB-expressing cells. Underlined values highlight the MIC values for quadruple compared to triple AcrB-expressing *E. coli* cells. *BZK* benzalkonium chloride, *CLX* cloxacillin, *ENO* enoxacin, *NOR* norfloxacin, *DEQ* dequalinium, *NB* novobiocin, *R6G* rhodamine 6G, *CV* crystal violet, *EtBr* ethidium bromide, *ACR* acriflavine, *MINO* minocycline, *EM* erythromycin, *RIF* rifampicin

**Fig. 2:**
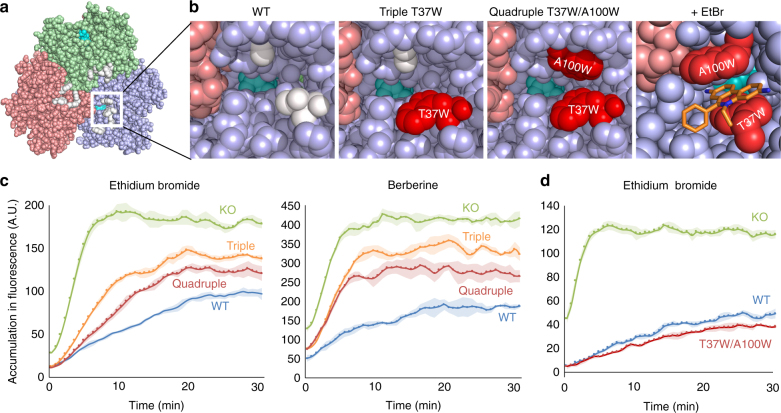
Effect of the combination of the T37W and A100W mutations on the EtBr and BER export activity. **a** Vertical upward view on the bottom of the porter domain of AcrB (the transmembrane domain is removed for a clear view). Green: access monomer, pink: extrusion monomer, blue: binding monomer. The mutated residues A33W, T37W, A100W and N298W are shown in white. The white box indicates the zoomed area used for the panels. **b** Zoomed images around the central cavity channel (CH3) entrance in the binding monomer, focused on the T37 and A100 mutation points. The DBP-bound drug minocycline is shown in cyan and is partially visible. T37W and A100W mutations are shown in red. Intercalated ethidium is shown in orange. Docking simulations were calculated by Glide (Schrödinger Suite)^42^. The images were created by PyMol. **c** and **d** Accumulation assays for wild-type and mutant AcrB. EtBr and BER fluorescence assays for triple (orange) and quadruple (red) mutant AcrB-expressing *E. coli* cells. Wild-type (WT, blue) and Δ*acrB* cells (KO, green) are also used as a control **c**. EtBr efflux assay for T37W/A100W double mutant (red) and wild-type (WT, blue) AcrB wild-type. Δ*acrB* results (KO) are shown in green **d**. Supplementary Fig. 5 also shows the export ability of T37W/A100W double mutant and wild-type AcrB. The average data points and the ± standard deviations shown by the error-envelopes are derived from four independent experiments (*n* = 4). The vertical axis shows the accumulation fluorescence in arbitrary units (A.U.). WT wild-type, KO *acrB*-knockout

When the A100W mutation was introduced to the triple mutant (creating the quadruple mutant A33W/T37W/A100W/N298W), the MIC for specific compounds increased one or two dilution steps compared to the triple mutant. This indicates a more active transport of these compounds by the quadruple mutant compared to the triple mutant AcrB. These compounds included benzalkonium (BZK), rhodamine 6G (R6G), ethidium bromide (EtBr) and acriflavine (ACR), which are all characteristic planar aromatic cations with a relatively low molecular mass. On the other hand, for the other compounds, the MIC was the same or decreased compared to the triple mutant. To understand this phenomenon, we looked at the locations of the Trp mutations in the central cavity. In the quadruple mutant (Fig. 2a), the planar aromatic cations can be intercalated between the parallel-arranged indole planes of T37W and A100W (Fig. 2b), facilitating the compounds’ movement through the entrance. As the N298W mutant AcrB was partially inactive, the addition of the T37W and A100W mutations could enhance the efflux of these cations in the impaired active AcrB quadruple mutant. The most significant increase in activity was seen for EtBr (two dilutions higher than triple, 512 µg ml^−1^ vs. 128 µg ml^−1^, with wild-type being 1024 µg ml^−1^) and BZK (one dilution higher than triple, 64 µg ml^−1^ vs. 32 µg ml^−1^, with wild-type being 256 µg ml^−1^).

The effect of the mutations was confirmed by liquid MIC (cell growth measurements in liquid broth instead of on agar plates) for three selected compounds: erythromycin (EM), EtBr and berberine (BER) (Table 2). Now, we also introduced CH2-mutations D566W and T678W, located at the lower cleft entrance connected to the periplasm (Fig. 1a and Supplementary Fig. 2a, b). Similar to the previous results, planar aromatic cations EtBr and BER showed a recovery of the MIC for the quadruple mutant compared to the triple mutant, while they were not affected by the CH2-mutations (except for the MIC for EtBr for the double CH2-mutant, which decreased only by one step). In sharp contrast, the MIC for EM decreased for the quadruple mutant compared to the triple mutant. More noticeably, the MICs for EM decreased significantly for both the single and double CH2-mutants by 1.5 and 4 dilution steps, respectively. The growth curves of these CH3-mutant AcrB-expressing cells in the presence of EM (Fig. 1c), EtBr (Fig. 1d) and BER (Fig. 1e) show the effect of the CH3-mutations on the transport ability of AcrB (other concentrations can be seen in Supplementary Fig. 4). The effect of these CH3-mutations on the cell growth in EM-supplemented broth was significantly smaller than those supplemented with EtBr or BER. The quadruple mutations negatively affected the growth in the presence of EM more than the triple mutations in contrast to the increased growth ability in the presence of EtBr and BER. In this case, the triple mutant-expressing cells showed no growth in 64 μg ml^−1^ EtBr, while the quadruple mutant-expressing cells grew significantly under the same conditions. Similarly, quadruple mutant-expressing cells were able to grow in 256 µg ml^−1^ BER, while the triple mutant-expressing cells were inviable. The quadruple mutant-expressing cells are able to grow in a significant one or two dilutions higher BER or EtBr concentration, respectively, compared to triple mutant-expressing cells (Table 2 and Supplementary Fig. 4). In addition, in the case of the CH2-mutations, the growth in the presence of EM was significantly decreased, especially in the case of the double mutant, while the growth in the presence of EtBr was hardly affected by the CH2-mutations compared to EM (Supplementary Figs. 2, 3). Even the double mutant-expressing cells showed sufficient growth in the presence of EtBr (Supplementary Fig. 3). Although the effect of the CH2-mutations on EtBr export was small, especially compared to the big impact on EM export, it is still possible that CH2 is used by planar aromatic cations, such as EtBr, which could explain the impact on EtBr efflux by the double tryptophan mutations. However, the results indicate that EM prefers CH2 to CH3, while planar aromatic cations such as EtBr prefer CH3. Recently, an uncertainty became apparent; the cleft entrance of CH2 may be hindered in the AcrAB−TolC tripartite efflux complex by the adaptor protein AcrA. On cryo-EM images of the AcrAB−TolC complex, the adaptor protein is attached to the cleft between subdomains PC1 and PC2^19–21^. However, the lower part of the cleft entrance can actively function as a substrate entrance, at least for drugs such as EM, considering the large effect of the CH2-mutations (even the single mutations) on the EM export activity.

**Table 2:** Liquid MIC values for CH3 and CH2 mutant AcrB expressing MG1655Δ*acrB E. coli* cells

	Strains and mutations	Minimal inhibitory concentration (MIC, µg ml^−1^)		
		PAC		HMMD
		BER	EtBr	EM
	Wild-type AcrB	512	512	512
	*acrB*-knockout	128	16	8
Channel 3	Triple (A33W/T37W/N298W)	**256**	**64**	**48**
	Quadruple (A33W/T37W/A100W/N298W)	**512**	**256**	**32**
	A100W	512	512	256
Channel 2	D566W	512	512	192
	T678W	512	512	192
	D+T (D566W+T678W)	**512**	**256**	**48**

Liquid MIC values are measured by a twofold dilution method by growing cells in LB broth supplemented with the drugs as described in Methods. Values are in µg ml^−1^. Bold values highlight the MIC values for CH3 triple, quadruple and CH2 AcrB-expressing *E. coli* cells. *BER* berberine, *EtBr* ethidium bromide, *EM* erythromycin, *PAC* planar aromatic cations, *HMMD* high-molecular-mass drug

The CH3-preferring compounds are all planar, aromatic and cationic compounds with a relatively low molecular weight (<500 Da). Examples of two HMMDs that showed decreased or similar MIC for quadruple compared to triple mutant AcrB were EM (~730 Da) and novobiocin (NB) (~610 Da), both uncharged and non-planar. The MICs are not increased (quadruple compared to triple) for small LMMDs, with a similar molecular weight as the CH3-preferring drugs, lacking a cationic charge, such as β-lactam cloxacillin (CLX) (~440 Da), fluoroquinolone norfloxacin (NOR) (~320 Da) and minocycline (MINO) (~460 Da). Also, cationic and aromatic antiseptic dequalinium (DEQ) (~460 Da) seems not to efflux more efficiently by the quadruple than the triple mutant, probably due its long size (a long alkyl chain between the cationic aminoquinaldine ends). This shows the specific characteristics of the CH3-preferring drugs and these are summarized in Supplementary Table 2.

### T37W/A100W mutations increase efflux for aromatic cations

To verify the role of the T37W and A100W mutations on the increased efflux of planar aromatic cationic drugs, we tested the efflux ability of triple, quadruple and T37W/A100W double mutant AcrB in more detail. Figure 2c shows the accumulation of EtBr and BER, measured by their fluorescence. These compounds emit fluorescence in bacterial cells by intercalating with DNA. When multidrug exporters are expressed and active in bacterial cells, the drugs are exported out of the cell before entering the cytoplasm. Therefore, the fluorescence signal is kept low. When there is no active transport, the fluorescence signal increases significantly. As shown in Fig. 2c, the EtBr and BER fluorescence level for the triple mutant was between the levels of *acrB-*knockout cells and wild-type AcrB-expressing cells, which indicates that the triple mutant has an intermediate transport activity for these compounds. The quadruple mutant shows a significantly lower level of fluorescence than the triple mutant, pointing to a recovery of the export activity by the introduction of the A100W mutation to the triple mutations for these planar aromatic cations, similar as seen in the plate and liquid MIC results discussed before. Especially for EtBr, the initial accumulation speed (the initial slope of the curve) is significantly lower for the quadruple compared to the triple mutant.

To further verify the effect of combining the T37W and A100W mutations at the CH3 entrance, we generated a T37W/A100W double mutant and compared its ability to extrude EtBr to that of the wild-type transporter. We found that the T37W/A100W double mutant AcrB exports EtBr more actively than wild-type AcrB. The rate of the accumulation of EtBr in the cells expressing the T37W/A100W double mutant AcrB is significantly lower than for cells expressing wild-type AcrB (Fig. 2d and Supplementary Fig. 5). The planar aromatic compound EtBr can thus be more effectively be extruded through CH3 by the increased substrate recognition due to the two indole planes at the entrance of the channel (Fig. 2b). T37W/A100W, triple and quadruple mutants of AcrB were expressed in similar abundance as wild-type AcrB (Supplementary Fig. 1).

### Competition of various drugs with ethidium bromide

**Fig. 3:**
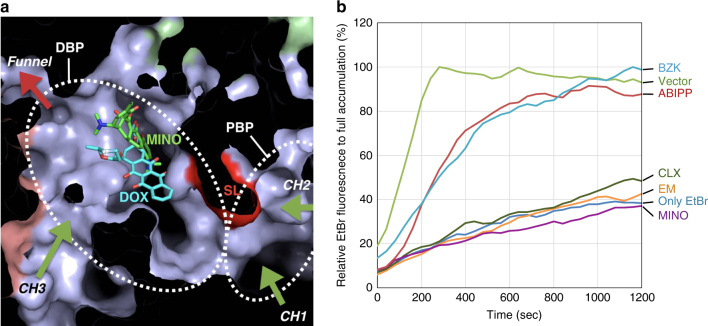
Competitive inhibition of several AcrB substrates on ethidium bromide efflux. **a** Cut side-view of the porter region of the binding monomer of AcrB, magnified around the drug-binding pockets and the intramolecular channels. DBP-bound minocycline (MINO, green sticks) and doxorubicin (DOX, cyan sticks) are superimposed. **b** Ethidium bromide (10 μm) competition fluorescence assay in combination with several substrates (100 μm) or the inhibitor ABI-PP (32 µg ml^−1^). The vertical axis shows the percentage of fluorescence compared to full accumulation in *acrB*-knockout *E. coli* cells. EM erythromycin, MINO minocycline, CLX cloxacillin, BZK benzalkonium. Shown is one of the results, repeats of the experiment gave similar results

The mutagenesis studies suggest that EtBr and other planar aromatic cations prefer CH3, while EM prefers CH2, which indicates that different routes are used by different drugs. To check the different routes of different drugs, we measured the competitive inhibition effects of various drugs on the EtBr efflux (Fig. 3b). The AcrB-specific inhibitor ABI-PP^22^ and BZK, a planar aromatic cation, inhibit the EtBr efflux completely when they were added in a 10 times higher molarity than EtBr. BZK was also exported more efficiently by the quadruple than the triple mutant-expressing cells, according to the MIC results (Table 1), which indicates that this compound is a CH3-preferring substrate as discussed earlier. However, the addition of the other drugs (CLX, EM and MINO) in a 10 times higher molarity had no effect on the EtBr efflux. These results indicate that the two CH3-preferring drugs EtBr and BZK are competing in the efflux cycle, while the other drugs are not competing with EtBr. Our previous study demonstrated that EM/RIF (rifampicin) and MINO/DOX (doxorubicin) bind in different binding pockets (the PBP and the DBP (Fig. 3a), respectively) and that DOX export is competitively inhibited by EM. This indicates that EM and DOX (and MINO) move through a common pathway^14^. The drug export is greatly decreased by mutations at both CH1 and CH2 as outlined in our previous study^14^ and current study (Supplementary Figs. 2, 3); therefore, the common route for these drugs is inferred to be CH1/CH2 and the PBP. In addition, CH2-mutations only had a minor effect on EtBr export activity (Supplementary Fig. 2). These combined findings suggest that CH3-preferring drugs and the other drugs use different pathways, which are not overlapping.

The originally postulated CH3 presented in our previous article^14^ is merged with the other channels to the PBP. However, here we found another possible pathway of CH3 that could be visualized in the binding monomer using CAVER software^23^. The original and the new CH3 open at a common site in the central cavity, although the new CH3 is branched and directed directly towards the DBP, as shown in Figs. 1a, 2a, b and 3a and Supplementary Fig. 9. Considering the results that PBP-binding drugs, such as EM, do not compete with CH3-preferring drugs, it can be presumed that the new CH3 directly connects to the DBP and is functioning. The CH3-preferring drugs thus bypass the PBP and the switch-loop. The new CH3 is connected to the DBP on the opposite side of the wall to which MINO and DOX are bound (Fig. 3a). It seems that planar aromatic cations can slip past the DBP-bound drugs without competition. It can be argued that the CH3-preferring drug export is not affected by the PBP-binding drugs, such as EM, because the CH3-preferring drugs do not go through the PBP.

### A switch-loop mutant can export CH3-drugs exclusively

In our previous study, we constructed the switch-loop mutant (SLM) G616P/G619P, in which switch-loop movement is prevented during functional rotation. The switch-loop is located between the PBP and DBP, and its swinging plays an important role in sending substrates from the PBP to the DBP by peristaltic motion. Therefore, the immobilized SLM almost completely lost its export activity^14^. However, since the CH3-preferring drugs do not go past the switch-loop and CH3 is connected to the DBP directly, there may be a possibility that a rigid SLM does not prevent the export of CH3-preferring drugs.

**Fig. 4:**
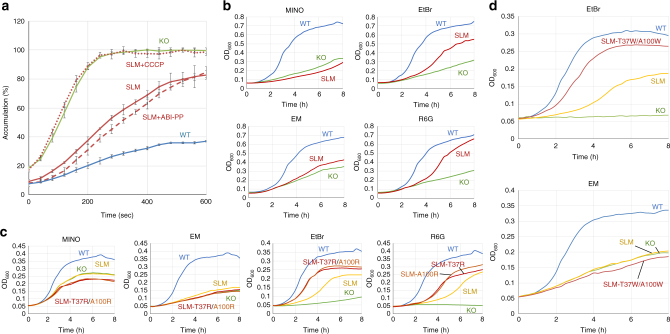
The effect of drugs and inhibitors on the export activity of the switch-loop mutant G616P/G619P AcrB (SLM). **a** EtBr fluorescence assay of SLM-expressing cells. Wild-type AcrB-expressing cells and *acrB*-knockout *E. coli* cells were also measured as a control. Blue, red and green lines indicate the wild-type AcrB-expressing, SLM AcrB-expressing and *acrB*-knockout *E. coli* cells, respectively. The dashed and dotted lines show the EtBr accumulation in SLM-expressing *E. coli* cells, under the addition of ABI-PP and CCCP, respectively. The average data points and the ± standard deviations are derived from three independent experiments (*n* = 3) and shown by the error bars. **b** The effect of several drugs on the growth of SLM-expressing *E. coli* cells (red). As a control, wild-type AcrB-expressing cells (blue) and *acrB*-knockout *E. coli* cells (green) were also used. **c** The effect of T37R and A100R mutations on the export of several drugs. Growth curves are provided for *acrB*-knockout (green), wild-type (blue), SLM (yellow), SLM-T37R (red) and SLM-A100R (orange) AcrB-expressing *E. coli* cells. **d** The effect of T37W/A100W double mutations on the export of EtBr and EM. Growth curves for *acrB*-knockout (green), wild-type (blue), SLM (yellow), SLM-T37W/A100W (red) AcrB-expressing *E. coli* cells are drawn. Abbreviations and concentrations used: SLM switch-loop mutant, MINO minocycline (0.125 μg ml^−1^), EM erythromycin (8 μg ml^−1^), EtBr ethidium bromide (8 in panels **c** and **d** or 16 μg ml^−1^ in panel **b**), R6G rhodamine 6G (8 μg ml^−1^). Shown is one of the results, repeats of the experiments gave similar results

Figure 4a shows the EtBr accumulation assay results. As expected, the G616P/G619P mutated SLM exhibited intermediate export activity for EtBr, with a fluorescence signal between those of wild-type AcrB-expressing cells and *acrB*-knockout cells. When carbonyl cyanide *m*-chlorophenylhydrazone (CCCP) (which dissipates the membrane potential and stops the energy supply for active drug exporters) was added, the accumulation of EtBr in the mutant-expressing cells was increased to the level of the *acrB*-knockout cells, indicating complete inhibition of active efflux of EtBr. Therefore, the intermediate accumulation of EtBr was entirely due to the SLM AcrB and suggests that an intermediate level of export remained. Interestingly, the inhibitor ABI-PP did not affect the intermediate level of activity of the SLM at all. In order for ABI-PP to inhibit the AcrB activity, ABI-PP must reach its binding site in the DBP^22^. The finding that ABI-PP fails to inhibit the export activity of the SLM indicates that ABI-PP does not pass the fixed switch-loop to reach its binding site, while EtBr is able to enter the DBP and is consequently exported out of the cell.

The ability of EtBr to be exported by the SLM was further confirmed by growth ability measurements of SLM-expressing cells in LB broth supplemented with MINO, EM, EtBr and R6G (Fig. 4b). Although limited growth ability was observed as expected, there was still a significant increase in growth ability for only EtBr and R6G when compared to *acrB*-knockout cells. However, this was not the case for MINO and EM. If the SLM AcrB is able to export only planar aromatic cations, the introduction of Thr37 and Ala100 central cavity residue mutations may alter the export of only these drugs. Therefore, we introduced arginine mutations in the SLM and tested the efflux ability of EtBr, R6G, EM and MINO again (Fig. 4c). We found that the SLM-T37R and SLM-A100R mutants export EtBr and R6G more efficiently than the mostly inactive SLM mutant. Cell growth of SLM-T37R and SLM-A100R AcrB-expressing cells was even possible in a one dilution higher EtBr and R6G concentration compared to SLM-expressing cells (Supplementary Fig. 7). On the other hand, for HMMD EM and LMMD MINO, the growth for SLM, SLM-T37R and SLM-A100R-expressing cells were identical to *acrB*-knockout cells, for all tested drugs concentrations. Supplementary Fig. 7 shows the growth curves for all tested concentrations of the drugs. All mutants were expressed equally compared to wild-type AcrB (Supplementary Fig. 1).

To further verify that CH3 is a route for planar aromatic cations from the central cavity to the DBP, we introduced the T37W/A100W double mutations to the SLM AcrB. As discussed previously, the T37W/A100W mutations significantly increase the efflux ability only for the planar aromatic cations (Figs. 1 and 2, Tables 1 and 2). The SLM mutant can export only the CH3-preferring planar aromatic cations, but not the LMMDs and HMMDs traversing CH1 and CH2 (Fig. 4b). If the SLM-T37W/A100W mutant of AcrB (in which the pathway from the PBP to the DBP is obstructed) can export CH3-preferring drugs more effectively than the SLM itself, it would indicate that planar aromatic cations can enter the DBP directly through CH3 from the central cavity without passing the PBP and the switch-loop. Figure 4d shows the growth curves of SLM and SLM-T37W/A100W AcrB-expressing *E. coli* cells. For EtBr, the T37W/A100W double mutations increase the export activity of the SLM mutant significantly. On the other hand, this was not the case for EM, where both the SLM and SLM-T37W/A100W mutant-expressing cells have the same growth ability as *acrB*-knockout cells. For EtBr, the SLM-T37W/A100W mutant-expressing cells are even viable at a one dilution higher EtBr concentration than the SLM mutant-expressing cells. Supplementary Fig. 8 shows the growth curves for all tested concentrations of EtBr and EM.

One of the main differences in chemical properties between EtBr/R6G and MINO is the cationic moiety, as they all have aromatic rings and a relatively low molecular mass (see Supplementary Table 2 for full comparison). This further indicates the importance of the cationic moiety in addition to the planar aromatic structure in order to go through CH3. Additionally, this shows that different pathways are used by drugs depending on their physicochemical properties and that specific drugs can enter the DBP directly via the central cavity of the AcrB trimer, from where they are exported through the exit funnel out of the cell.

## Discussion

The results presented above indicate that different pathways are used by drugs with different physicochemical properties. Planar aromatic cations such as EtBr, BZK, BER and R6G seem to primarily go through CH3 from the central cavity and enter the DBP directly, bypassing the PBP and the switch-loop. Although CH3 is open in the binding monomer, CH3-preferring drugs can already be positioned in the central cavity at the access monomer, while the CH1 and CH2-preferring drugs are located in the PBP. For example, DBP-bound drug DOX (which is loosely present in the PBP at the access stage before being translocated to the DBP) or PBP-bound drug EM^11, 14^. The export of CH3-preferring drugs was not inhibited by CH1 and CH2-preferring drugs, but only by the CH3-preferring drug BZK (Fig. 3b). Additionally, the SLM fixated mutant can still exclusively actively export CH3-preferring planar aromatic cations (Fig. 4 and Supplementary Figs. 7, 8). The SLM combined with the T37R or A100R mutations can exclusively export planar aromatic cations significantly more effectively than the SLM itself (Fig. 4c and Supplementary Fig. 7). The quadruple (T37W/A100W-containing) mutant has a significantly higher export activity for the characteristic CH3-preferring drugs compared to both triple (N298W containing) and N298W mutant AcrB (Tables 1 and 2, Figs. 1 and 2c and Supplementary Fig. 4), indicating that the T37W and A100W mutations restore the partially inactive N298W mutant by the increased substrate binding between the tryptophan indole planes. Indeed, the T37W/A100W double mutant is able to export EtBr significantly more efficiently even than wild-type AcrB (Fig. 2d and Supplementary Fig. 5). Furthermore, the SLM-T37W/A100W mutant has a significantly increased export activity for EtBr, but not at all for EM (Fig. 4d and Supplementary Fig. 8). CH2 tryptophan mutations have a significant impact on the export of EM (Table 2, Supplementary Figs. 2, 3). The CH2 mutations also affect the EtBr efflux ability slightly, which could be explained by the possibility that planar aromatic cations also go through CH2 and that the tryptophan mutations sterically hinder EtBr (Supplementary Fig. 3). The finding that planar aromatic cations prefer CH3 from the central cavity directly to the DBP can also explain the relatively minor inhibitory effect of competitive-type efflux pump inhibitors such as PAβN on EtBr efflux^24^.

CH3-preferring drugs are planar, low in molecular mass, have aromatic rings and a cationic nitrogen in their aromatic moiety (Supplementary Table 2). These small planar aromatic cationic compounds may be able to diffuse through the Thr37 and Ala100 tryptophan double mutated CH3 by cation-π and π−π interactions between the substrate and the tryptophan residues (Fig. 2b), perhaps similar to the cation–π interactions in the binding of methylated GDP in translation-initiation factor eIF4E^25^. Cation−π interactions also play an important role in the recognition of similar substrates found in this research (e.g. EtBr and BZK) in the staphylococcal multidrug transporter Smr^26^ by conserved residues Tyr59 and Trp62^27, 28^. In multidrug-resistance regulator RamR, π−π interactions between Phe155 and aromatic cations EtBr, BER, DEQ, R6G and crystal violet also play an important role in recognition and binding^29^. Our T37W/A100W double mutant is able to efflux EtBr significantly more effectively than wild-type AcrB (Fig. 2d and Supplementary Fig. 5) and the SLM-T37W/A100W mutant can export EtBr significantly more effectively than the SLM itself (Fig. 4d and Supplementary Fig. 8), which suggests that the two parallel tryptophan indole planes increase the binding and recognition of these CH3-compounds in the central cavity. Planar aromatic cations can move into the hydrophobic environment between the tryptophan indole moieties.

A recent study discovered that an I38F mutant increases the susceptibility to EtBr and BER^30^ where Ile38 is located next to the Thr37 residue, also supporting the finding that these drugs prefer CH3. Furthermore, maleimide**-**labelling in AcrB suggests that the vestibules and central cavity play a role in substrate translocation^16^. It was also found that an RND transporter in a Gram-positive organism could export EtBr, which could indicate that the central cavity might play a role in the export of planar aromatic cationic drugs from the cytoplasm^31^. In addition, central cavity mutations in homologue EmhB of *Pseudomonas fluorescens* also negatively affected the MICs for certain drugs^32^, which suggests that the central cavity plays a role in the drug extrusion process. We also found that replacing Thr37 and Ala100 with a charged hydrophilic arginine enhances the efflux in the SLM significantly for the charged planar aromatic cations (Fig. 4c and Supplementary Fig. 7). The introduction of the very hydrophilic arginine residues probably stimulates the efflux of the cationic CH3-preferring drugs by a significantly increased hydrophilic channel entrance rather than rejecting the cationic drugs. Perhaps, the negatively charged Glu130 and Asp174 residues and the four polar Ser(132–135) residues (which lie in between the entrance of CH3 and the DBP, facing the inside of the channel) can help these cationic drugs traverse CH3 directly towards the DBP. We found that mutations of the residues Glu130 and Asp174 increased the susceptibility (although slightly) for EtBr, but did not affect the efflux activity for EM (Supplementary Fig. 6). However, this effect was small and the exact mechanism of translocating through the AcrB channels requires additional investigation.

Substrates of which the antimicrobial action occurs in the cytoplasm, such as EtBr, are actively transported from the cytoplasm to periplasm by other transporters^33–35^ and then exported through the outer membrane by RND transporters. Recently, a third drug-binding site in the transmembrane groove between TM1 and TM2 was found by crystallography^36^, which suggests a transmembrane flopping pathway of AcrB for cytoplasmic substrates. The groove binding site is close to the entrance of CH1 and the vestibule connecting to CH3. Perhaps substrates flipped from the cytoplasm use the CH1 or CH3 pathways, while CH2 may function as a periport entrance.

In summary, we proved that CH3 plays a role in the translocation of planar aromatic cationic low-molecular-mass substrates, but not for other compounds, e.g. LMMD minocycline and HMMD erythromycin. Although multiple potential substrate channels of AcrB have been identified^11, 12, 14–16^, here we showed that different substrates use different channels depending on their physicochemical properties. This knowledge should be taken into account for the development of new antibiotics. The significance of AcrB having multiple channels lies in its broad substrate specificity.

## Methods

### Bacterial strains and growth conditions

For the minimal inhibition concentration experiments, the *E. coli* MG1655 strain^37^ was used as the wild-type strain and ∆*acrB* (NKE96)^38^ and ∆*tolC* (NKE95)^39^ were derived from MG1655. In addition, BL21 strains were used for the efflux assays. Gene deletion was performed according to the method of Datsenko and Wanner, with recombination between short homologous DNA regions catalysed by phage **λ** Red recombinase^40^. The drug resistance markers were eliminated using plasmid pCP20^40^. The bacterial strains were grown at 37 °C in Luria-Bertani broth^41^.

### Site-directed mutagenesis

The plasmid pBAD33acrB (pBAD33 carrying the *acrB*-gene from MG1655, including a C-terminal hexahistidine-tag) was used for the site-directed mutagenesis. Primers were used to introduce the mutations (Supplementary Table 1) by polymerase chain reaction. The mutations were confirmed by sequencing. Plasmids were transformed in *acrB*-deficient MG1655 (for MIC) or *acrB*-deficient BL21 (for efflux assay) *E. coli* cells. The triple mutant was created originating from the pBAD33acrB(A33W/T37W) plasmid. The quadruple mutant was created from the triple mutant plasmid. The SLM plasmid used for site-directed mutagenesis was isolated from the NKE1620 *E. coli* stock^14^.

### Potential AcrB channel discovery and analysis

The mutation sites were chosen after analysis of possible channels drawn by CAVER^23^ software (using starting coordinates residue 37 from each monomer (centre of the central cavity), using probe radius of 1.2 Å, shell radius of 3.0 Å, shell depth of 4.0 Å and AcrB crystal structural data from e.g. PDB accession number 3AOD (CAVER software version 3 was used)). Similar channels could be seen in other crystal structures of AcrB. A channel connecting the PBP was found in the access monomer^14^. The bottleneck size of the tunnel was estimated to be 2.2 Å. The channels found in the binding monomer were connected to the PBP (this channel is connected to the pockets right behind the switching-loop) and DBP, with a bottleneck of 1.6 and 2.0 Å, respectively. These two tunnels share the same entrance to the central cavity. A loop of the residues 131–134 exists between the tunnels. There is third branched channel that connects to the DBP from this shared entrance space. Using CAVER software with starting residues 37 and 100 (CH3 entrance) and residues 130, 291 and 615 (DBP) from the binding monomer (using probe radius of 1.2 Å, shell radius of 2.0 Å, shell depth of 4.0 Å and AcrB crystal structural data from e.g. PDB accession numbers 3AOD or 4DX5) gave us all channels (CH1, CH2 and CH3) and the binding pockets (PBP and DBP) and are shown in Supplementary Fig. 9. Although there are three channels sharing the same entrance of CH3 to the DBP given by the CAVER software, we chose the CH3 connected directly to the DPB in the binding monomer as the main possible channel, taking into account the finding that CH1 and CH2-preferring compounds are not competitively inhibiting the efflux of the CH3 drug ethidium bromide (Fig. 3).

### Drug susceptibility by MIC

The minimal inhibitory concentrations (MIC) values were determined using LB-agar plates or by growth ability in liquid LB supplemented with substrates, in a series of dilutions. Cell cultures of MG1655∆*acrB* cells harbouring the plasmid of interest were grown overnight, stamped on agar plates (containing 10 mm arabinose) and incubated overnight at 37 °C. Alternatively, liquid cultures (supplemented with 10 mm arabinose) were incubated, shaken at 37 °C and OD_600 nm_ readings were performed by using the Infinite M200 Pro (Tecan). MIC values were defined as the lowest drug concentrations at which the cells were not viable anymore.

### Drug efflux assay

For the ethidium bromide and berberine efflux assays, plasmids were isolated from the MG1655-strains and transformed into *acrB*-deficient BL21 *E. coli* cells. Overnight cultures of the *E. coli* cells were diluted and grown at 37 °C until OD_600 nm_ 0.5–0.6 was reached. Cells were harvested and washed twice with 5 ml Efflux Buffer (100 mm potassium phosphate (pH 7.5) and 5 mm MgSO_4_) and diluted to final OD_600 nm_ of 36. Substrates were added to a final concentration of 10 µm. Other substrates (erythromycin, minocycline, cloxacillin and benzalkonium) were added to 100 µm to create a 1:10 molar ratio. The final ABI-PP concentration was 32 µg ml^−1^. Cells were supplemented with 10 mm arabinose during measurements. Ethidium bromide fluorescence was measured by SH-8100 reader (Corona Electric Co.) using *λ*_ex_  = 530 nm and *λ*_em_  =  600 nm. For berberine, *λ*_ex_  =  363 nm and *λ*_em_  =  530 nm was used. Efflux assays were repeated at least three or four times providing the ± standard deviations shown by the error-envelopes.

### Data availability

The data that support the findings of this study are available from the corresponding author upon request.

## Electronic supplementary material

Supplementary Figure
