# Supplementary material for: Multiple entry pathways within the efflux transporter AcrB contribute to multidrug recognition
Source: Nat Commun. 2018 Jan 9;9:124. doi: 10.1038/s41467-017-02493-1 (PMC5760665; doi:10.1038/s41467-017-02493-1)
Supplement: Supplementary file 1 — Supplementary Figure [file 41467_2017_2493_MOESM1_ESM.pdf]

Supplementary Information

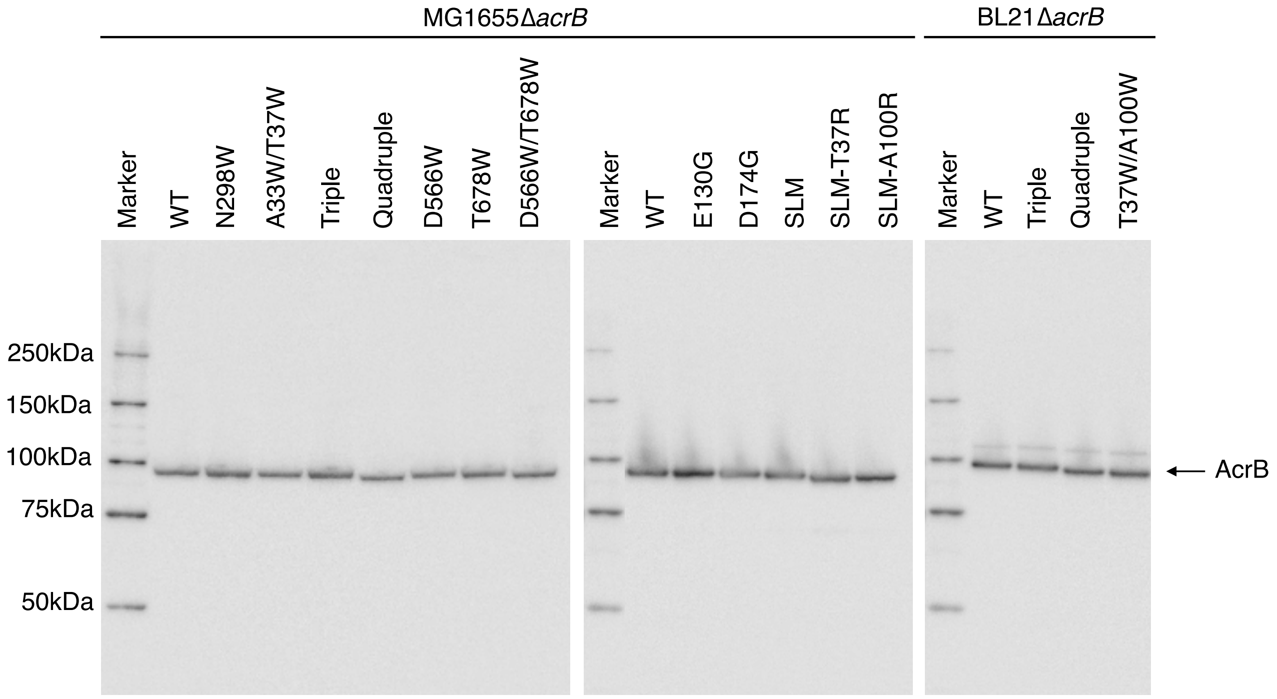

Supplementary Figure 1. Western blotting results of mutant AcrB. Wild-type and mutant AcrB were expressed in MG1655Δ*acrB* and BL21Δ*acrB* from pBAD33 plasmids. Membrane factions of AcrB expressing cells were harvested and run on a 10% SDS gel (2μg protein) and transferred to a PVDF membrane. Western blotting was performed using an anti-polyhistidine antibody for the first reaction and mouse IgG HRP-linked antibody for the second reaction.

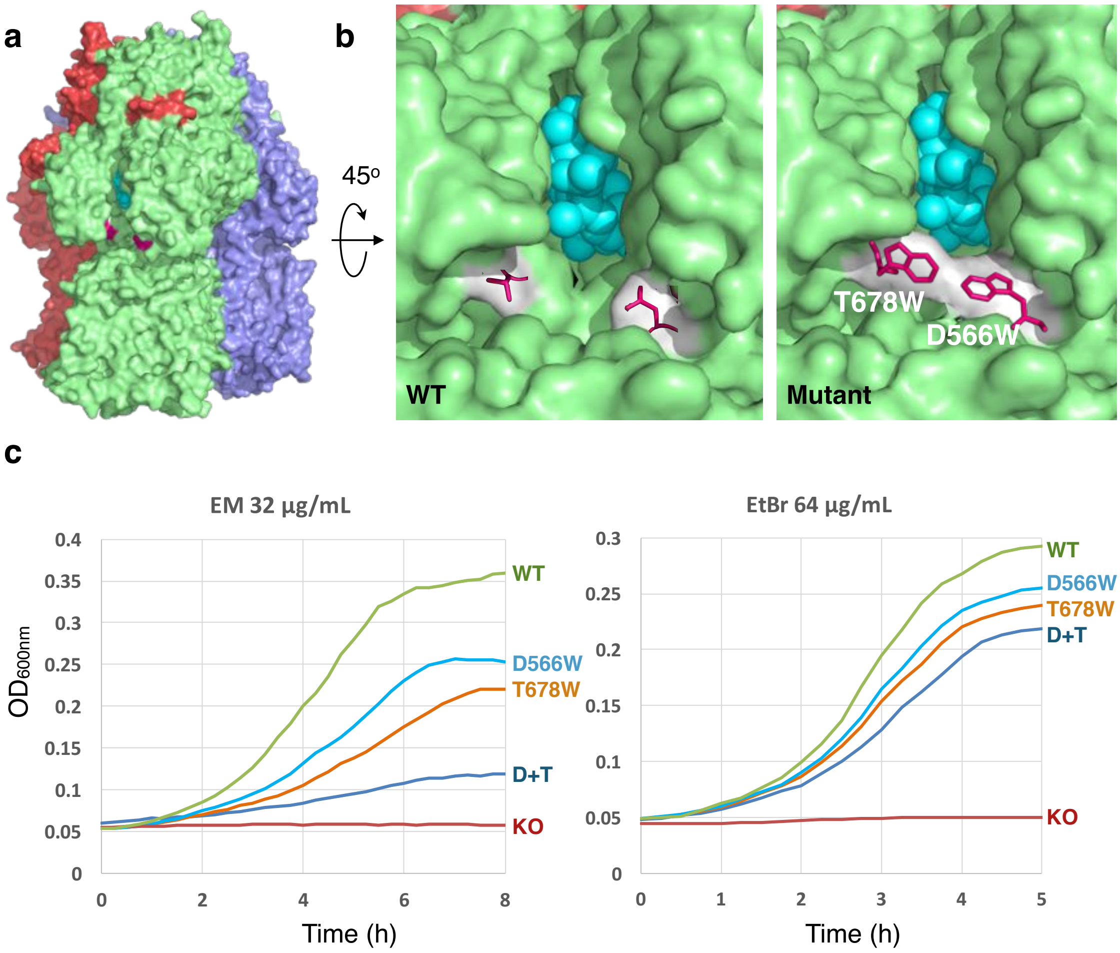

Supplementary Figure 2. The effect of CH2 mutations on the efflux ability of EM and EtBr. The single and double D566W and T678W mutations are located in the lower cleft region. **a)** Purple indicates the mutation-site. Green shows the access monomer with rifampicin bound and depicted in cyan. **b)** Close-up view of the mutation-site. The white highlighted area with the purple sticks shows the locations of position 566 and 678 in wild-type (left) and double mutant (right) AcrB. **c)** Growth ability of *acrB*-knockout, wild-type and single and double mutant expressing *E. coli* cells. Abbreviations and concentrations used: EM, erythromycin (32µg/ml); EtBr, ethidium bromide (64µg/ml). Complete sets of the growth curves (for all concentrations) of CH2 mutant expressing cells are shown in Supplementary Fig. 3.

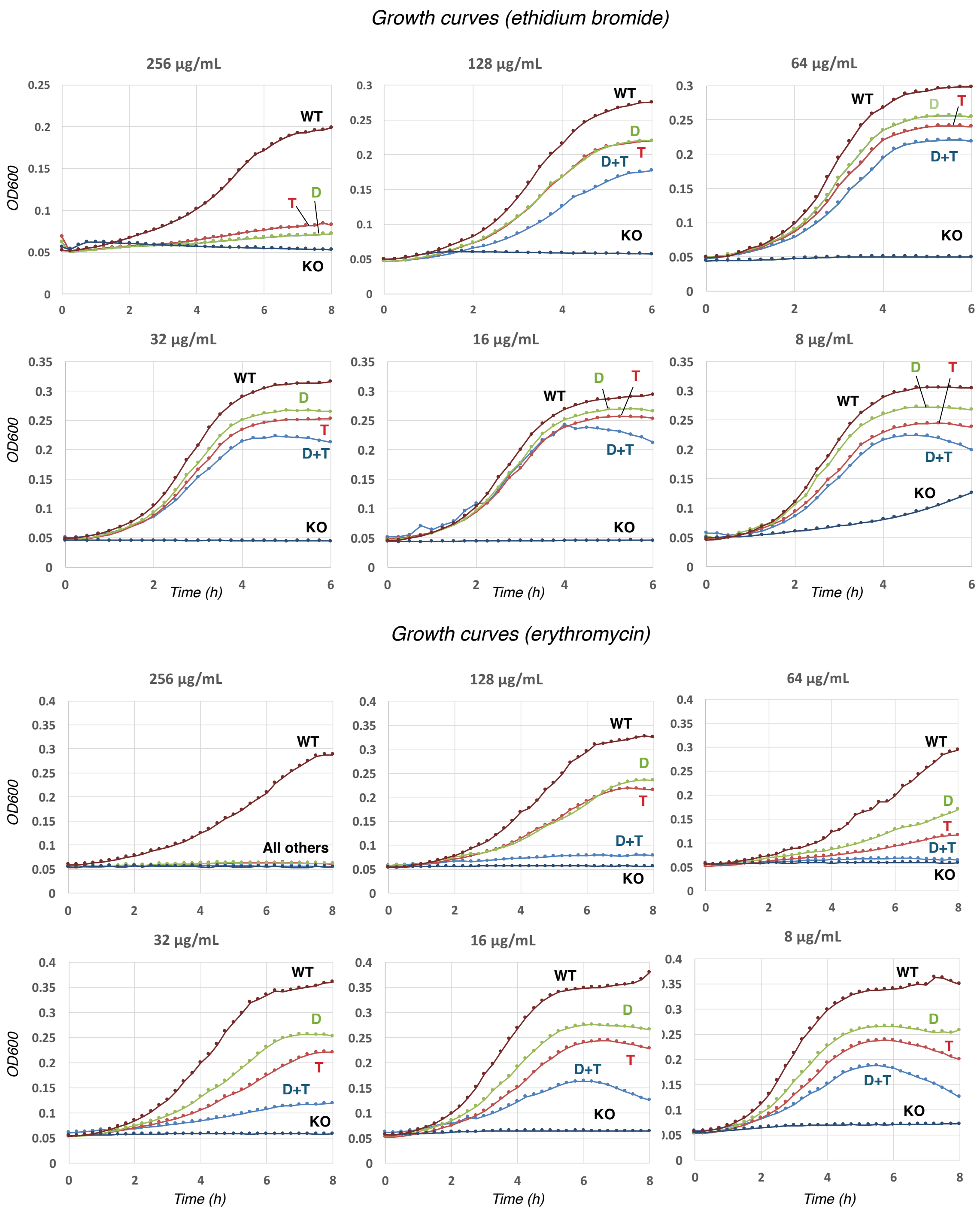

Supplementary Figure 3. The effect of CH2 mutations on the export activity of AcrB for EtBr and EM. Growth ability of *acrB*-knockout (KO), wild-type (WT), single CH2 D566W (D) and T678W (T), and double CH2 (D+T) mutant AcrB-expressing cells, combined in one graph. A 2-fold stepwise gradient of compounds was used ranging from 512 µg/mL to 8 µg/mL (note: all cells were completely inviable in 512 µg/mL for both EtBr and EM). Shown is one of the results, repeats of the experiment gave similar results. Abbreviations: EM, erythromycin and EtBr, ethidium bromide.

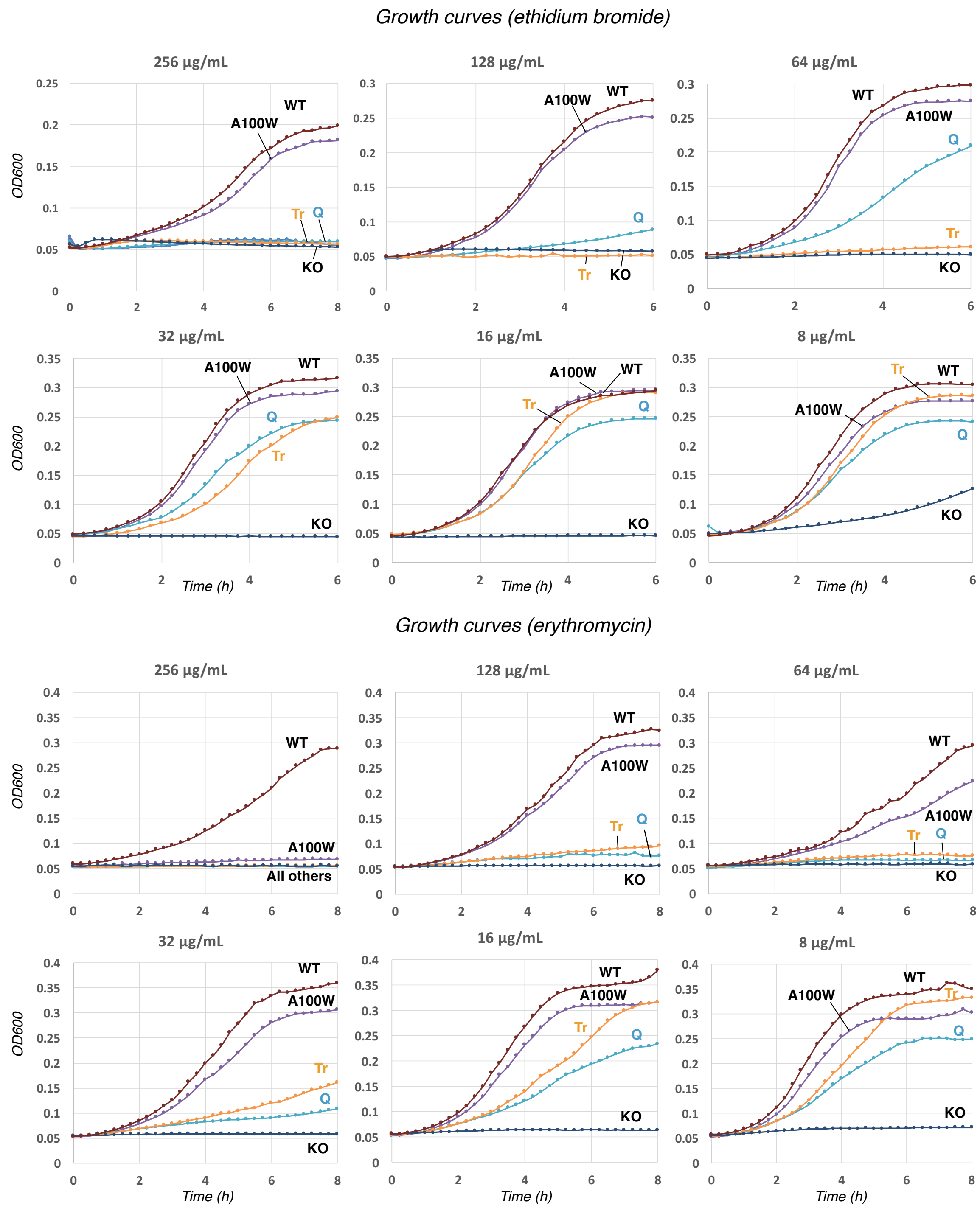

Supplementary Figure 4. The effect of CH3 mutations on the export activity of AcrB for EtBr and EM. Growth ability of *acrB*-knockout (KO), wild-type (WT), A100W, triple CH3 (Tr) and quadruple CH3 (Q) mutant AcrB-expressing cells, combined in one graph. A 2-fold stepwise gradient of compounds was used ranging from 512 μg/mL to 8 μg/mL (note: all cells were completely inviable in 512 μg/mL for both EtBr and EM). Shown is one of the results, repeats of the experiment gave similar results. Abbreviations: EM, erythromycin and EtBr, ethidium bromide.

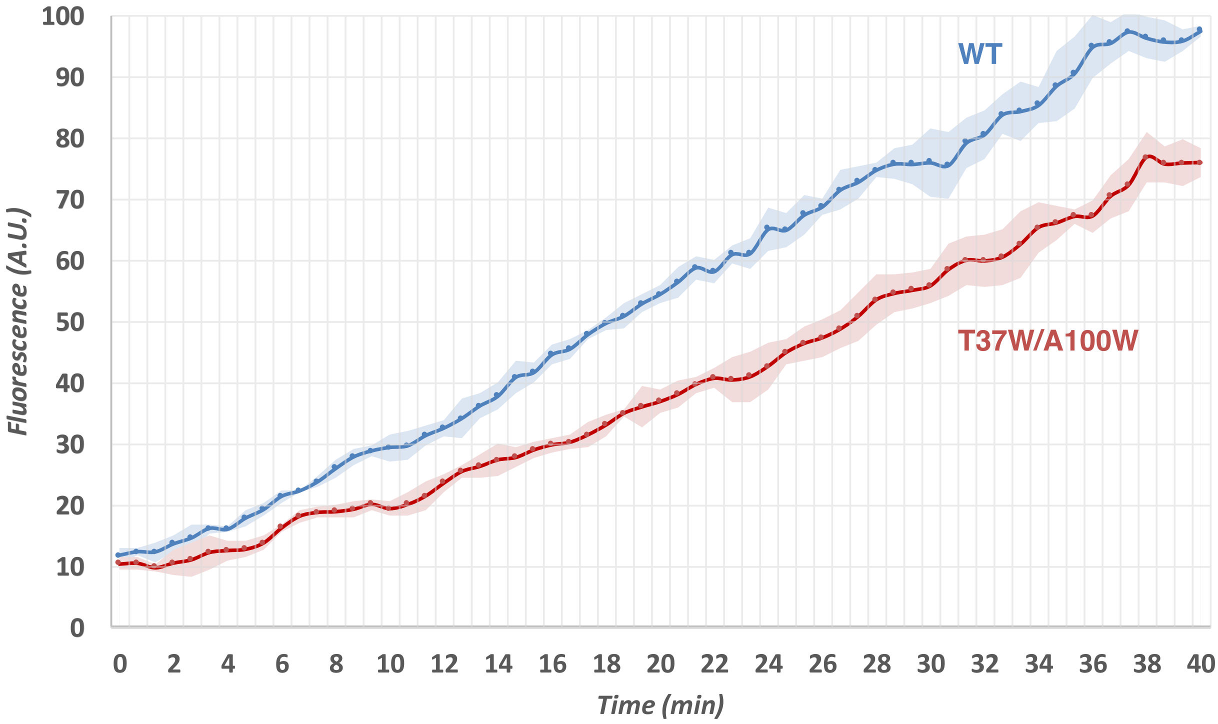

Supplementary Figure 5. Ethidium bromide efflux ability of the T37W/A100W double mutant AcrB. EtBr accumulation assay for wild-type and T37W/A100W double mutant (red) and wild-type (WT, blue) AcrB expressing. The average data points and the ± standard deviations are derived from four independent experiments (n=4) and shown as error envelopes. Vertical axis shows fluorescence in arbitrary units (A.U.). Abbreviation: WT, wild-type.

Growth curves (ethidium bromide)

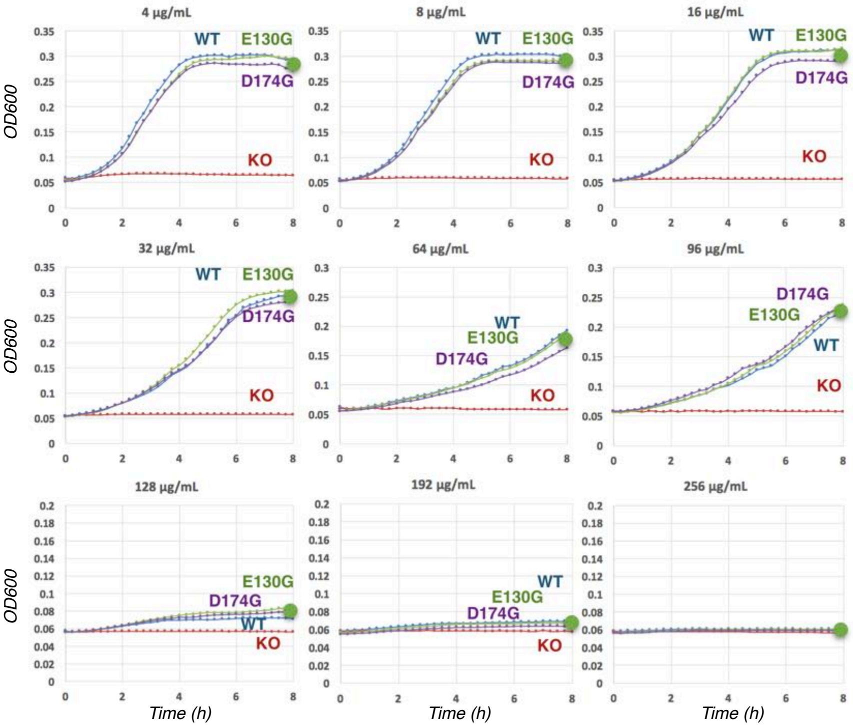

Growth curves (ethidium bromide)

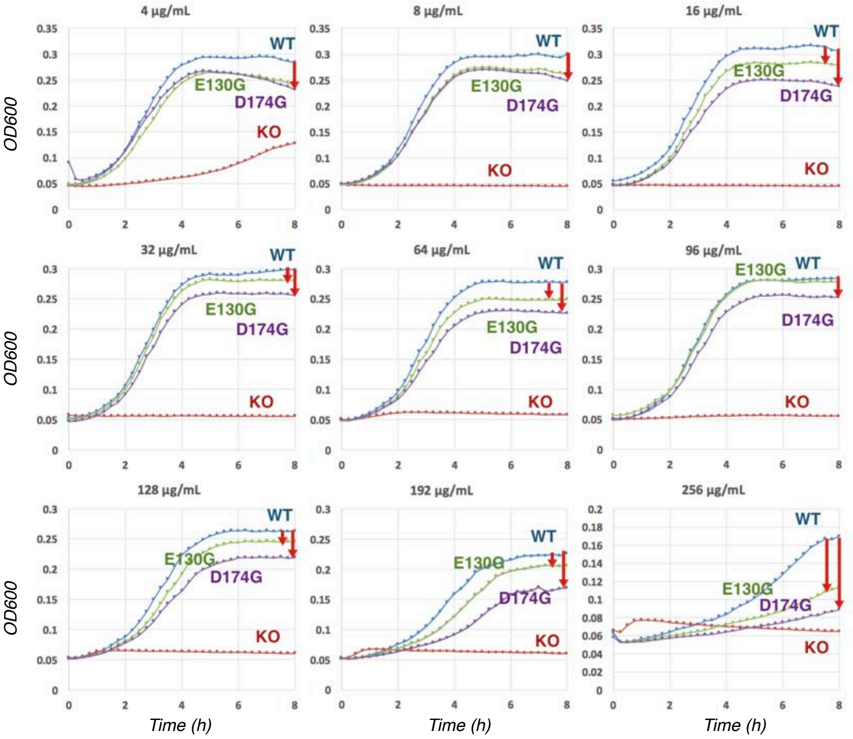

Supplementary Figure 6. A possible role of the negatively charged CH3 Glu130 and Arg174 residues on the efflux of cationic compounds. Growth ability of *acrB*-knockout, wild-type (blue) and CH3 Glu130 (green) and Arg174 (purple) glycine-mutant AcrB expressing *E. coli* cells (*acrB*-knockout cells (red) were also used as a control). Broth was supplemented with erythromycin or ethidium bromide in a step-wise gradient. (note: all cells were completely inviable in 512 µg/mL for both ethidium bromide and erythromycin). Shown is one of the results, repeats of the experiment gave similar results. Abbreviations: WT, wild-type and KO, *acrB*-knockout.

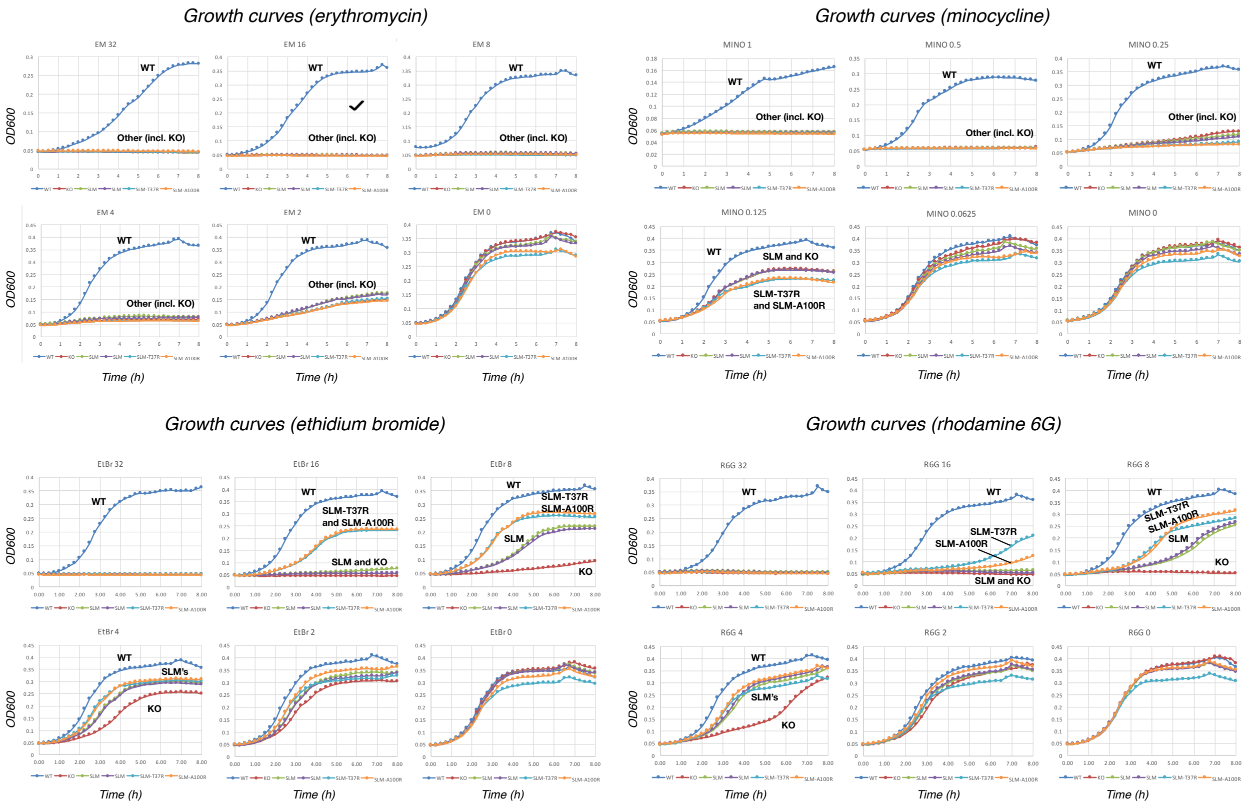

Supplementary Figure 7. The effect of T37R and A100R mutations on the export activity of SLM AcrB for EtBr, R6G, EM and MINO. Growth ability of SLM mutants under a 2-fold dilution series of several compounds (EtBr and R6G: planar aromatic cations, MINO: low-molecular-mass-drug, EM: high-molecular-mass-drug). Shown are *acrb*-knockout cells (red) and wild-type (dark blue), SLM (green and purple), SLM-T37R (light blue) and SLM-A100R (orange) AcrB expressing cells. Abbreviations: SLM, switch-loop mutant; MINO, minocycline; EM, erythromycin; EtBr, ethidium bromide; R6G, rhodamine 6G.

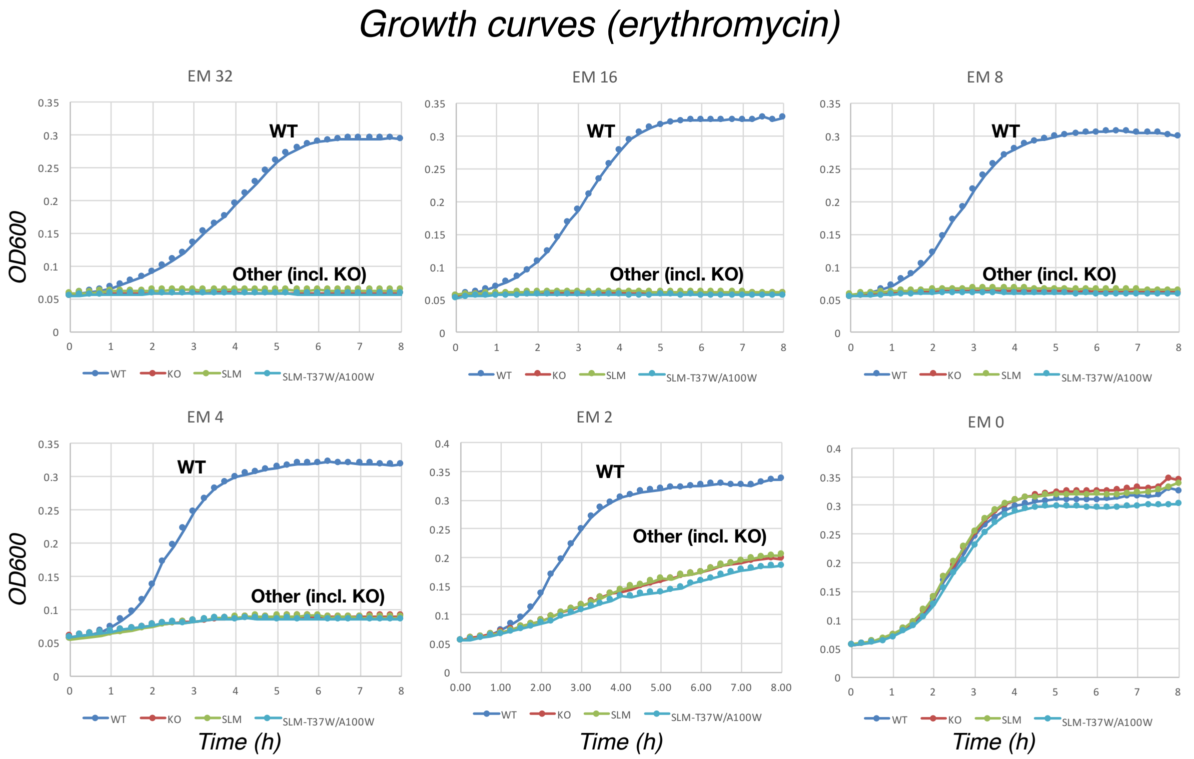

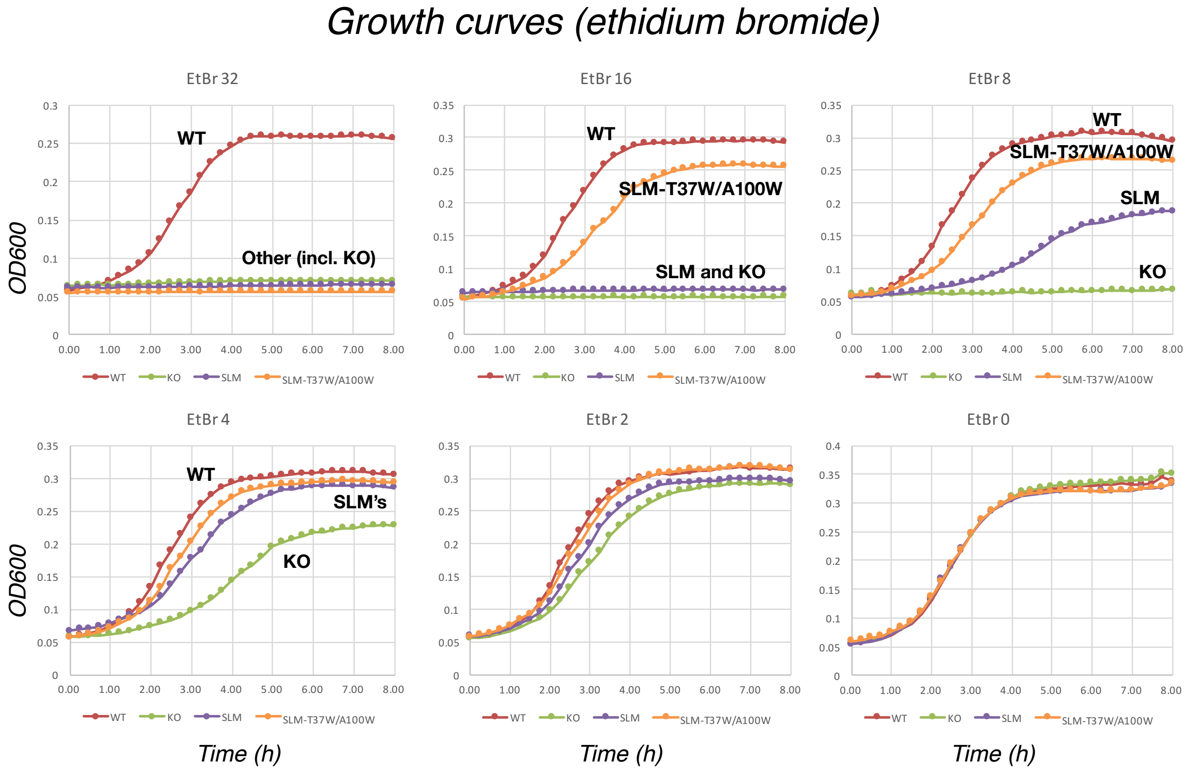

Supplementary Figure 8. The effect of T37W/A100W double mutations on the export activity of SLM AcrB for EtBr and EM. Growth ability of SLM mutants under a 2-fold dilution series of several compounds. Shown are *acrb*-knockout cells and wild-type, SLM and SLM-T37W/A100W AcrB expressing cells. Abbreviations: SLM, switch-loop mutant; EM, erythromycin; EtBr, ethidium bromide.

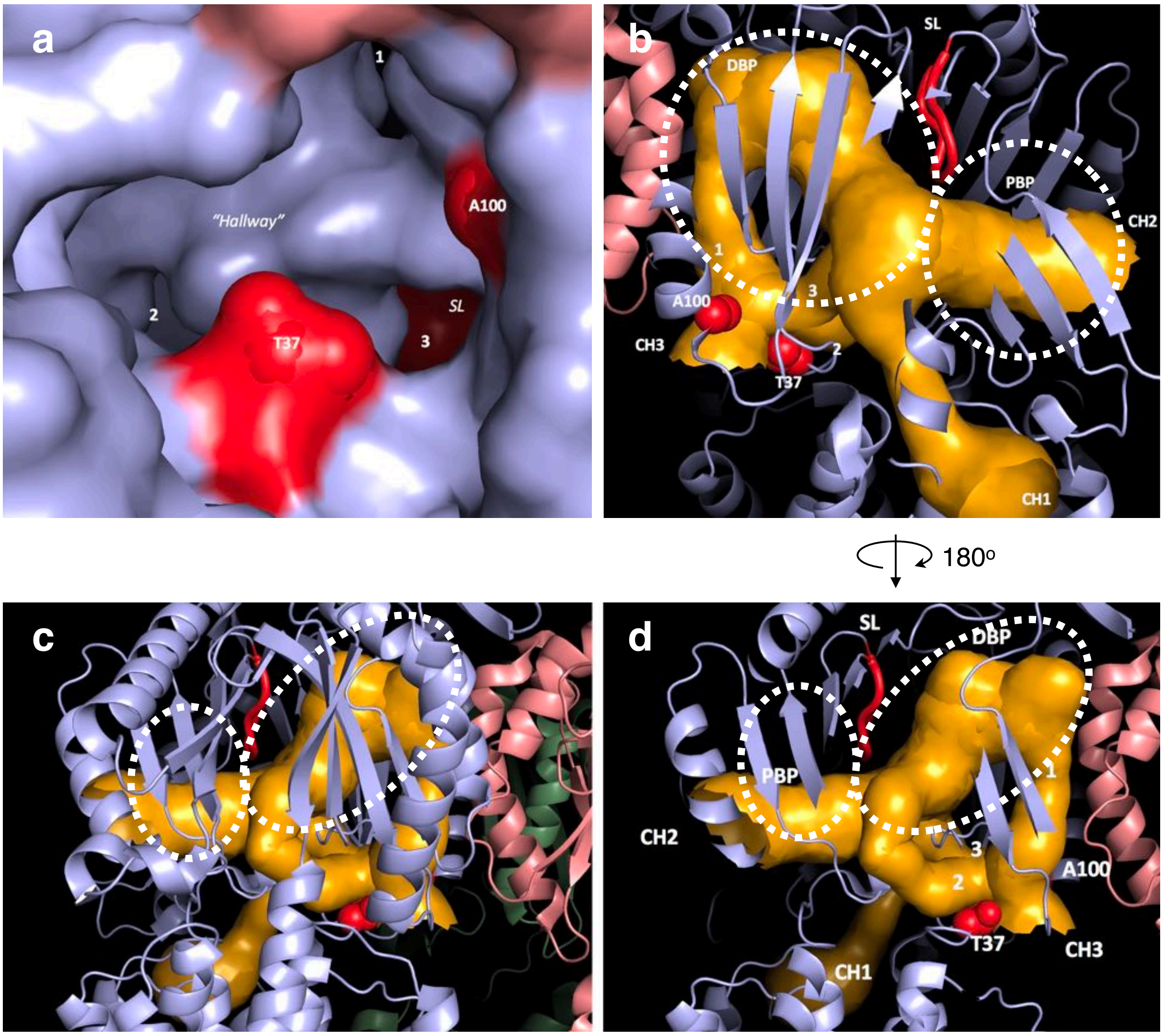

Supplementary Figure 9. Possible tunnels by CAVER leading to the binding pockets in the AcrB multidrug exporter. Three tunnels of CH3 were calculated to exist to connect the central cavity to the distal binding pocket (DBP) in the binding monomer of AcrB. Membrane surface channel CH1 and periport channel CH2 are also drawn. All tunnels are visualized in orange and calculated by using PDB 4DX5 (high-resolution structure of AcrB at 1.9Å). The tunnels are separated by a loop existent of the residues Ser(132-135).

Supplementary Table 1. Primers used for site-directed mutagenesis.

Primer name	Codon alteration	Primer sequence
AcrB_E566W_fwd	GAT ⟶ TGG	TTCTTGCCATGGGAGGACCAGGGCGTGTTT
AcrB_E566W_rev		CTGGTCCTCCCATGGCAAGAAGGAGCTTGG
AcrB_T678W_fwd	ACC ⟶ TGG	GGTACTGCATGGGGCTTTGACTTTGAGCTG
AcrB_T678W_rev		TCAAAGCCCCATGCAGTACCCAGTTCCAC
AcrB_T37W_fwd	ACG ⟶ TGG	TATCCTTGGATTGCACCGCCGGCAGTA
AcrB_T37W_rev		TGCAATCCAAGGATATTGCGCCACCGG
AcrB_A100W_fwd	GCG ⟶ TGG	ACTGATTGGGATATCGCGCAGGTTCAG
AcrB_A100W_rev		GATATCCCAATCAGTACCAGACTCAAA
AcrB_A33W_T37W_fwd	GCG ⟶ TGG, ACG ⟶ TGG	CCGGTGTGGCAATATCCTTGGATTGCA
AcrB_A33W_T37W_rev		ATATTGCCACACCGGCAGTTTGAGGAT
AcrB_N298W_fwd	AAC ⟶ TGG	GGTGCATGGGCGCTGGATACCGCTGCG
AcrB_N298W_rev		CAGCGCCCATGCACCGGTCGCCAGCTT
AcrB_E130G_fwd	GAG ⟶ GGC	AGCGTTGGCAAATCATCCAGCAGCTTC
AcrB_E130G_rev		TGATTTGCCAACGCTCACCCCTTGCTG
AcrB_D174G_fwd	GAT ⟶ GGC	GTGGGTGGCGTTCAGTTGTTCGGTTCA
AcrB_D174G_rev		CTGAACGCCACCCACGCCCGACGTACG
AcrB_T37R_fwd	ACG ⟶ CGT	TATCCTCGTATTGCACCGCCGGCA
AcrB_T37R_rev		TGCAATACGAGGATATTGCGCCAC
AcrB_A100R_fwd	GCG ⟶ CGT	ACTGATCGTGATATCGCGCAGGTT
AcrB_A100R_rev		GATATCACGATCAGTACCAGACTC

Primers were designed by using the software Genetyx (Genetyx Co.). Tryptophan mutations were created by replacing the codons of interest to TGG, glycine mutations to GGC and arginine mutations to CGT (see primer sequence). Generally, 9 nucleotides upstream and 18 downstream of the codon of interest were selected for primer-design.

Supplementary Table 2. Overview of physicochemical properties of several compounds.

	Group	Substrate	MW (Da)	LogP	Volume (Å3)	TPSA (A^2)	Aromatic rings	Charge	Structure
**ACR**	PAC	Acriflavine	224	-1.79	210	56	3	**Cation** x1	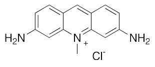
**EtBr**	PAC	Ethidium bromide	314	0.3	298	56	4	**Cation** x1	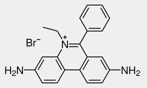
**NOR**	LMMD	Norfloxacin	319	-0.69	279	75	1	0	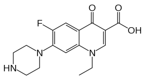
**ENO**	LMMD	Enoxacin	320	-0.38	275	87	1	0	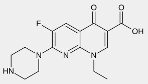
**BZK**	PAC	Benzalkonium (C_{14})	333	4.45	333	385	1	**Cation** x1	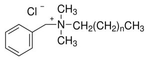
**BER**	PAC	Berberine	336	0.2	296	41	3	**Cation** x1	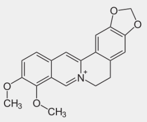
**CV**	PAC	Crystal violet	373	3.2	378	10	3	**Cation** x1	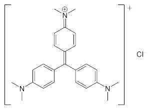
**CLX**	LMMD	Cloxacillin	436	2.66	350	113	1	0	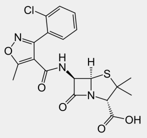
**R6G**	PAC	Rhodamine6G	444	3.95	425	65	3	**Cation** x1	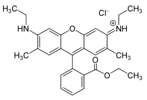
**DEQ**	LMMD	Dequalinium	457	0.11	465	60	4	**Cation** x2	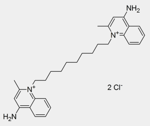
**MINO**	LMMD	Minocycline	457	-0.23	399	165	1	0	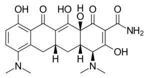
**NB**	HMMD	Novobiocin	613	3.93	538	200	2	0	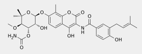
**EM**	HMMD	Erythromycin	734	2.28	709	194	0	0	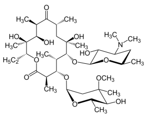
**RIF**	HMMD	Rifampicin	823	2.62	756	220	2	0	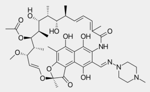

The designated Group, LogP, molecular weight (Da), volume (A^3), surface charge (TPSA, Å^2), aromatic rings and charge for the compounds used in this study. Abbreviations: PAC, planar aromatic cations; LMMD, low-molecular-mass drug; HMMD, high-molecular-mass drug.
